# Benchmarking Deep Learning for NSCLC PET/CT Segmentation on a Histologically Confirmed Vietnamese Dataset: Validation and Generalization

**DOI:** 10.3390/jimaging12080364

**Published:** 2026-08-08

**Authors:** Quang Tuan Ho, Ngoc Ha Bui, Thuy Duong Tran, Quang Huy Khuat, Ngoc Toan Tran, Xuan Chung Le, Huu Quyet Nguyen, Tat Thang Nguyen, Van Thai Nguyen, Dinh Thuy Mai, Quang Duy To, Dinh Chau Nguyen, Nguyen Huong Giang Trinh, Van Chinh Cao, Tien Hung Bui, Thu Trang Vu, Khac Nam Vo, Hai Quan Ho

**Affiliations:** 1 Institute for Nuclear Science and Technology, Hanoi 100000, Vietnam; tuanhq.11@gmail.com (Q.T.H.);; 2 Faculty of Engineering Physics, Hanoi University of Science and Technology, Hanoi 100000, Vietnam; 3 Department of Radiation Oncology and Radiosurgery, 108 Military Central Hospital, Hanoi 100000, Vietnam; 4 Department of Medical Imaging Technology, Hanoi Medical College, Hanoi 100000, Vietnam; 5 Hong Ngoc-Phuc Truong Minh General Hospital, Hanoi 100000, Vietnam; 6 Faculty of English, Thuong Mai University, Hanoi 100000, Vietnam; 7 Nuclear Medicine Department, HCM Oncology Hospital, Ho Chi Minh City 700000, Vietnam; 8 Viettel Institute for Strategic Technology, Viettel Group, Hanoi 100000, Vietnam

**Keywords:** NSCLC, PET/CT, deep learning, nnU-Net, domain shift, transfer learning, tumor segmentation

## Abstract

Accurate segmentation of non-small cell lung cancer (NSCLC) on positron emission tomography/computed tomography (PET/CT) is an essential prerequisite for automated metabolic tumor volume (MTV) quantification and staging. Although deep learning models achieve high performance on large-scale datasets, their generalization across different clinical domains is limited by variations in imaging protocols and patient demographics. This study aims to evaluate several deep learning architectures and investigate a transfer learning strategy to mitigate domain shift. Three architectures, including ResNet-backbone 3D U-Net, nnU-Net v2, and Swin UNETR, were benchmarked from scratch and compared with a fine-tuned nnU-Net initialized with AutoPET II weights. Results on the internal dataset showed that the fine-tuned nnU-Net achieved a Dice similarity coefficient (DSC) of 83.4 ± 6.5%, a 95% Hausdorff distance (HD95) of 5.1 ± 3.6 mm, and a precision of 89.6 ± 8.2%. Compared to the nnU-Net v2, the fine-tuned nnU-Net improved the absolute DSC by 6.8% while reducing local training time by 37.5% by bypassing the initial feature-learning phase. The fine-tuned nnU-Net model also demonstrated a high correlation between the MTV and the ground truth (Pearson *r* = 0.96, *p* < 0.001), indicating its potential as a reliable automated approach for quantitative MTV extraction and NSCLC prognostic-related analysis.

## 1. Introduction

Non-small cell lung cancer (NSCLC) is a leading cause of cancer-related mortality globally, with an increasing incidence in Asian countries, including Vietnam. In modern clinical diagnostics, ^18^F-FDG positron emission tomography/computed tomography (PET/CT) plays an important role in tumor staging and treatment response assessment by combining anatomical details from CT with metabolic characteristics from PET. This multimodal integration can improve diagnostic accuracy by 20% to 30% compared to standalone CT [1]. However, manual 3D tumor segmentation remains a challenge: it is time-consuming, highly subjective, and suffers from inter-observer variability [2]. Such inconsistencies reduce the reliability of quantitative biomarkers like metabolic tumor volume (MTV) and total lesion glycolysis (TLG), which are essential for prognosticating patient outcomes [3].

The application of deep learning, particularly self-configuring architectures like nnU-Net, has established new high-performance baselines in medical image segmentation [4]. Recent international challenges, such as AutoPET, have demonstrated that automated models can achieve a Dice similarity coefficient (DSC) of approximately 0.80 on large-scale datasets [5,6]. Despite these advancements, generalization remains limited when deploying these models across different clinical environments. The performance of PET-based segmentation algorithms is influenced by domain shift, which originates not only from variations in patient demographics and pathological characteristics but also from differences in imaging hardware, acquisition protocols, and reconstruction methods across institutions [7].

Furthermore, the lack of histologically confirmed datasets in contemporary AI research limits the assessment of clinical accuracy [8]. While several large-scale public PET/CT datasets exist (e.g., AutoPET, LUNG-PET-CT-Dx), they often suffer from an absence of comprehensive histological validation or remain unoptimized for evaluating domain shift across diverse ethnicities [8]. To address these limitations, our study introduces and benchmarks a large-scale dataset comprising 400 PET/CT scans from a Vietnamese patient cohort [9]. The primary contributions of this study are: (1) histopathological confirmation of all NSCLC cases; (2) a multi-rater annotation protocol involving three experienced physicians, combined with the STAPLE consensus algorithm [10,11]; and (3) the implementation of preprocessing pipelines to improve the tumor-to-background signal ratio.

Utilizing this dataset enhances model reliability and improves demographic representation in global medical AI development [12,13]. Specifically, this study benchmarks contemporary reference algorithms on the Vietnamese PET/CT dataset. Furthermore, domain shift and cross-generalization capabilities are evaluated using international public datasets [6,14]. Finally, the correlation between AI-predicted volumetric metrics and clinical outcomes is analyzed in accordance with recent biomedical evaluation guidelines [15].

## 2. Materials and Methods

**Figure 1:**
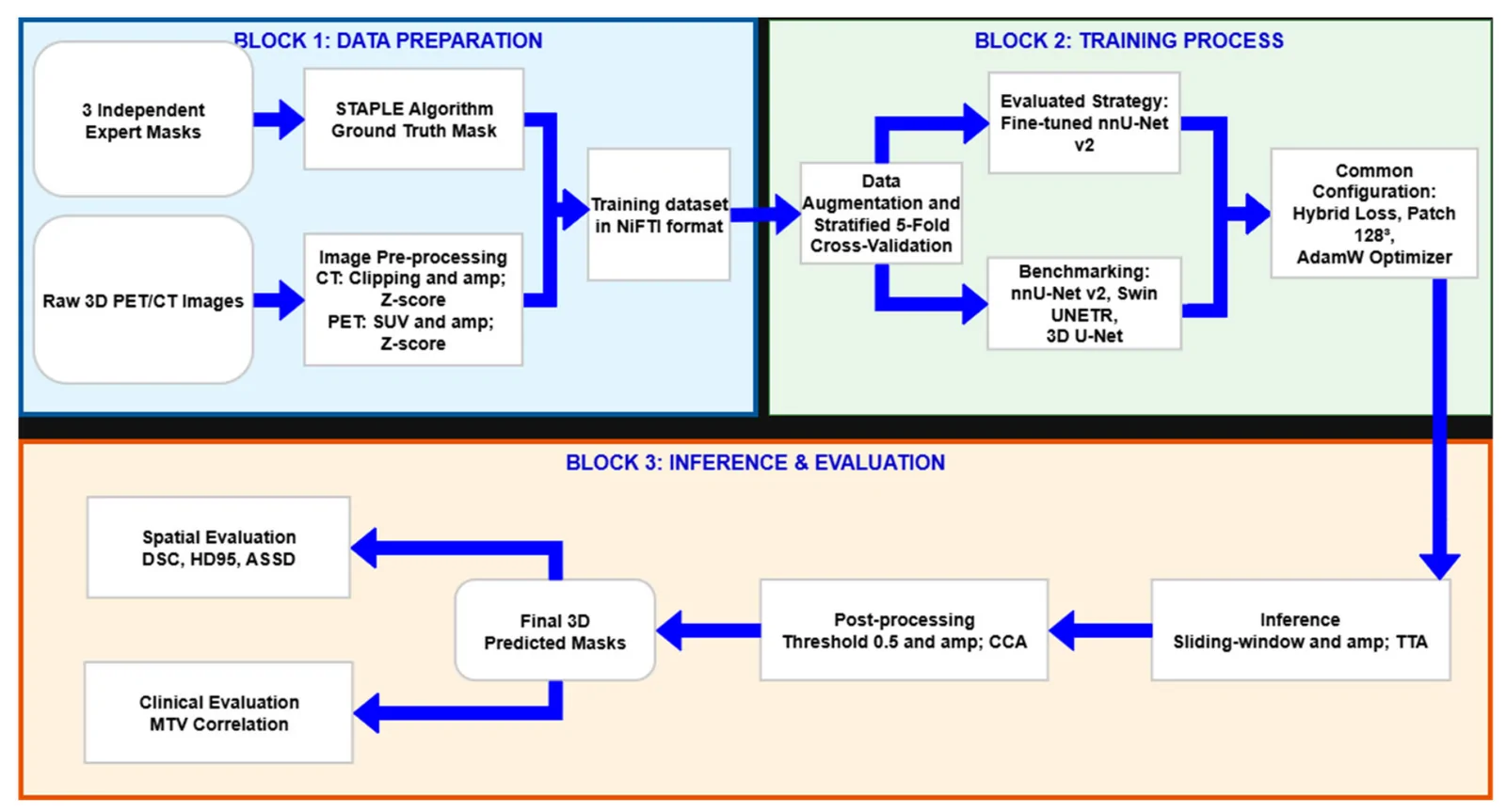
Overview of the implemented validation end-to-end framework for automated NSCLC segmentation.

The automated NSCLC segmentation framework is illustrated in Figure 1. The evaluated pipeline operates in three sequential steps. First, during the data preparation, raw 3D PET/CT images are pre-processed and a consensus ground truth is generated from three expert annotations using the STAPLE algorithm, followed by conversion into NIfTI format. Second, during the training phase, the dataset is partitioned using stratified 5-fold cross-validation. An nnU-Net v2 model is fine-tuned and compared against models trained from scratch (standard nnU-Net v2, Swin UNETR, and ResNet-backbone 3D U-Net) under a unified configuration (hybrid loss, patch size of 128 × 128 × 128, AdamW optimizer). Finally, in the inference and evaluation stage, predictions are generated using a sliding-window strategy with test-time augmentation, followed by post-processing to obtain final 3D segmentation masks. Model performance is assessed using spatial metrics (DSC, HD95, ASSD) and clinical relevance via MTV correlation.

### 2.1. Dataset Preparation

This study utilized an extended version of the Vietnamese NSCLC PET/CT dataset [9]. Data were collected between June 2019 and June 2023 from three oncology centers: Vietnam National Cancer Hospital, Hanoi Oncology Hospital, and Ho Chi Minh City Oncology Hospital. Clinicopathological characteristics of the patient cohort and PET/CT dataset are shown in Table 1. A total of 416 whole-body PET/CT scans were initially retrieved. After excluding 16 cases with respiratory motion artifacts, 400 cases were included for analysis. This cohort consisted of 284 NSCLC patients (with a total of 342 primary and metastatic lesions, averaging 1.2 lesions per patient) and 116 negative controls (healthy individuals or patients with benign pathologies). The negative controls were included to evaluate the model’s capacity to control the false-positive rate in regions with physiological FDG uptake, analogous to the design of the AutoPET dataset. All 284 NSCLC cases were confirmed via histopathology (biopsy or surgery).

To address the imaging domain characteristics, the acquisition parameters across the participating centers were standardized. Following standard clinical preparation, patients were instructed to fast for at least 6 h prior to the scan, and blood glucose levels were verified to be within normal limits. ^18^F-FDG was administered intravenously with a weight-based activity ranging from 3.7 to 5.5 MBq/kg, followed by a standardized uptake time of approximately 60 min.

Whole-body PET/CT scans were primarily performed using Siemens Biograph mCT systems (Siemens Healthineers, Erlangen, Germany) across the Vietnam National Cancer Hospital, Hanoi Oncology Hospital, and Ho Chi Minh City Oncology Hospital. Unenhanced low-dose CT scans were acquired for attenuation correction and anatomical localization. The native CT images were reconstructed with a matrix size of 512 × 512 pixels, an in-plane pixel spacing of 1.037 × 1.037 mm^2^, and a slice thickness of 3.26 mm. For the PET imaging, data were acquired in 3D mode and reconstructed using the ordered-subset expectation maximization (OSEM) algorithm with 2 iterations and 24 subsets, resulting in a matrix size of 192 × 192 pixels, an in-plane resolution of 3.646 × 3.646 mm^2^, and a matching slice thickness of 3.26 mm.

**Figure 2:**
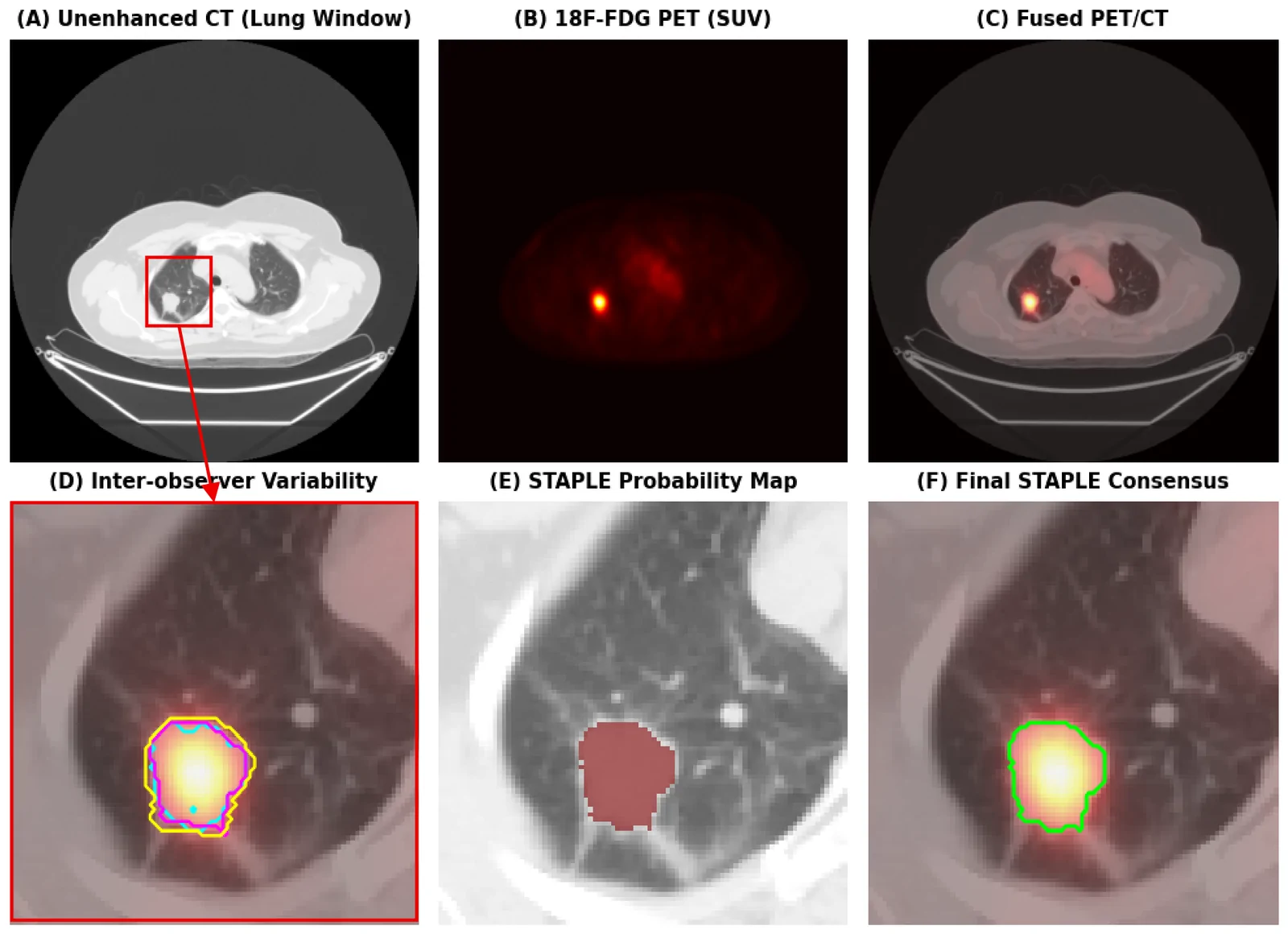
Visualization of multimodal PET/CT data and the ground-truth generation process. (**A**) Unenhanced CT; (**B**) ^18^F-FDG PET (SUV map); (**C**) Fused PET/CT; (**D**) Inter-observer variability among three experts; (**E**) STAPLE probability map; and (**F**) Final probabilistic consensus used as the reference standard.

Figure 2 illustrates the multimodal imaging inputs and the ground-truth generation workflow utilized in this study. By fusing anatomical details from unenhanced CT scans with metabolic activity from ^18^F-FDG PET, NSCLC lesions were localized and subsequently manually delineated in 3D Slicer (version 5.2.2; Surgical Planning Laboratory, Brigham and Women’s Hospital, Boston, MA, USA) by three senior nuclear medicine physicians, each with over 10 years of clinical experience in oncologic PET/CT imaging. To account for inter-observer variability, the STAPLE algorithm [10] was applied to synthesize these independent annotations into a unified probability map, which was then thresholded at *p* ≥ 0.5 to produce a binary reference standard based on the optimal weighting of each rater’s performance. This process yields a consistent consensus that mitigates subjective human bias [11], providing a reference standard for training the deep learning architectures. Finally, the dataset was partitioned using a 5-fold cross-validation strategy, stratified by tumor volume and SUVmax, and standardized into 3D binary masks (where 1 represents the tumor region and 0 denotes healthy tissue).

### 2.2. Image Preprocessing

The entire preprocessing pipeline was implemented via the nnU-Net v2 framework (version 2.2.1) [4]. To harmonize the native heterogeneous spatial resolutions described above (CT: 1.037 × 1.037 × 3.26 mm^3^ vs. PET: 3.646 × 3.646 × 3.26 mm^3^), both PET and CT images were uniformly resampled to an isotropic voxel spacing of 2.0 × 2.0 × 2.0 mm^3^. Cubic spline interpolation was used for intensity data, and nearest-neighbor interpolation was applied for mask labels to preserve the multi-rater STAPLE tumor boundaries.

- CT Channel: Window clipping was applied at [−1000, 400] HU to focus on the lung parenchyma and exclude bone artifacts. Subsequently, Z-score normalization was applied. Following the default and validated workflow of the nnU-Net v2 architecture, the normalization statistics (mean and standard deviation) were automatically extracted from the global foreground voxels of the entire training directory (imagesTr) prior to cross-validation fold splitting. This standard procedure establishes a stable intensity baseline across the dataset without introducing spatial feature leakage.
- PET Channel: Radioactivity intensity was converted to Standardized Uptake Value (SUV) based on body weight and clipped at [0, 20] to mitigate the impact of outliers. Similar to the CT channel, the PET Z-score normalization was executed using the global dataset-level foreground statistics as defined by the default nnU-Net v2 preprocessing pipeline.

Data augmentation was applied dynamically (on-the-fly) during training using the *batchgenerators* framework, rather than expanding the dataset offline. Each 3D patch sampled for a batch was subjected to stochastic transformations (including random rotation, scaling, and Gaussian noise simulation) with a predefined probability of 20% per transformation.

### 2.3. Segmentation Models

The selection of deep learning architectures in this study aims to optimize segmentation performance and establish a benchmarking system to evaluate the adaptability of contemporary models to the specific anthropometric characteristics and image distributions of Vietnamese patients. The nnU-Net v2 was utilized as the baseline model due to its automated self-configuring capabilities based on data characteristics, allowing for the determination of training parameters without manual intervention. With an input patch size of 128 × 128 × 128 voxels, this model simultaneously ingests multi-channel PET and CT data, facilitating consistent delineation of anatomical boundaries [16].

To address the limitations of traditional CNNs, which are constrained by local receptive fields, the Swin UNETR architecture was included to evaluate the self-attention mechanism of the Transformer network. By tokenizing 3D data into patches and processing them through hierarchical Swin Transformer blocks with a 7 × 7 × 7 window size, this model is capable of modeling the long-range dependencies of biomedical signals. This characteristic is suitable for identifying large NSCLC tumors or those with complex invasive morphologies, contributing to segmentation sensitivity [17].

Finally, a ResNet-backbone 3D U-Net [18] based on the MONAI framework (version 1.3.0) [19] was included as an architectural baseline to assess the efficacy of feature fusion at the encoder level. The performance contrast between this model and the fine-tuned nnU-Net version indicates that standard architectures, when not subjected to transfer learning on the target data domain, may exhibit limited capability in mitigating domain shift.

### 2.4. Training and Implementation Details

To ensure a standardized benchmark, the experimental setup was divided into shared environmental constraints and architecture-specific optimization schedules. All models were trained on an identical hardware environment equipped with an NVIDIA RTX Pro 6000 Blackwell Max-Q Edition GPU (96GB GDDR7; NVIDIA Corporation, Santa Clara, CA, USA). The shared constraints included a multi-channel input patch size of 128 × 128 × 128 voxels, a synchronized dynamic patch extraction mechanism, and the hybrid loss function.

Patch extraction was performed during the training process. To mitigate the class imbalance between the NSCLC tumors and the background, a forced oversampling strategy was employed. Specifically, one-third of the extracted patches within each training batch were constrained to center on a foreground class (tumor voxel), while the remaining two-thirds were sampled uniformly at random across the entire 3D image volume. Furthermore, the total number of patches extracted per original image is not a fixed integer; instead, patches are continuously and stochastically sampled across the entire training dataset to formulate 250 training iterations per epoch.

Training regimen:

Optimization schedules were tailored to achieve convergence for each specific network topology rather than enforcing a fixed epoch budget. For the nnU-Net architecture, the training process adhered to the default schedule with a maximum of 1000 epochs. Notably, the fine-tuned nnU-Net model was initialized with weights from the AutoPET II 2023 challenge (publicly shared on the Zenodo platform by Isensee et al. [20]) and fine-tuned for 500 additional epochs. Algorithmic convergence was defined by the exhaustion of the polynomial learning rate (polyLR) schedule, wherein the learning rate incrementally decayed to near zero. Network weights were selected based on the highest exponential moving average of the pseudo-Dice score evaluated on the validation set to prevent overfitting. For architectures deployed via the MONAI framework (ResNet-backbone 3D U-Net and Swin UNETR), the models were trained for 300 epochs using the AdamW optimizer, reaching convergence via an early-stopping mechanism. The initial learning rate was set at 1 × 10^−4^ with a cosine annealing decay schedule and a weight decay coefficient of 1 × 10^−5^. The overall batch size was established at 8 through a gradient accumulation mechanism.

Loss function:

A hybrid loss function was utilized to optimize the network, combining the stability of the cross-entropy loss with the capability of handling class imbalance inherent to the soft-Dice loss. In this evaluated framework, the weighting coefficients for both loss components were set to a 1:1 ratio. This equal weighting follows the empirically validated default settings of the nnU-Net architecture. Maintaining this 1:1 ratio balances the voxel-level classification from the cross-entropy loss and the spatial overlap optimization from the Dice loss, which facilitates model convergence without requiring manual hyperparameter tuning. The formula is defined as follows:

(1) $$L=L_{CE}+L_{Dice}$$

in which the components are calculated according to the formula:

(2) $$L_{CE}=-\frac{1}{N}\sum_{i=1}^{N}y_{i}\log(p_{i})$$

(3) $$L_{Dice}=1-\frac{2\sum_{i=1}^{N}y_{i}p_{i}+\varepsilon }{\sum_{i=1}^{N}y_{i}+\sum_{i=1}^{N}p_{i}+\varepsilon }$$

where $y_{i}$ is the actual ground truth label at the *i*-th voxel; $p_{i}$ is the predicted probability of the model at that voxel; *N* is the total number of voxels in the 3D image space; and ε is a very small smoothing constant to prevent division by zero errors.

Inference and Post-processing:

The inference process on the test set applied a sliding-window approach with a 50% patch overlap, combined with test-time augmentation (TTA) techniques by averaging predictions across image mirroring operations. The post-processing step utilized a connected component analysis (CCA) algorithm to eliminate small noise regions, retaining lesion clusters. An internal ablation study indicated that combining a probability threshold of 0.5 with TTA and CCA reduced the false-positive area in regions of physiological FDG uptake (such as brown fat and myocardium).

### 2.5. Evaluation Metrics

The evaluation of spatial accuracy and detection performance followed guidelines from Metrics Reloaded [15]. To ensure computational reproducibility, metric evaluations were executed via the monai.metrics library with explicit parameter configurations. Specifically, the 95% Hausdorff Distance was calculated using *HausdorffDistanceMetric* configured with *percentile* = 95 and *include_background = False*. The Average Symmetric Surface Distance (ASSD) was computed using *SurfaceDistanceMetric* with *symmetric = True* and *include_background = False*. For all distance-based metrics, the physical voxel spacing was automatically extracted from the NIfTI affine matrix to account for the actual physical dimensions (mm) rather than voxel counts. The mathematical formulations are defined as follows:

- DSC: Measures the degree of spatial overlap between the predicted label (P) and the ground truth (G).

(4) $$DSC=\frac{2\left|P\cap G\right|}{\left|P\right|+\left|G\right|}$$

- HD95: Computes the maximum distance between two sets of surface points while excluding the top 5% of outliers, thereby mitigating noise caused by isolated, highly divergent predictions.

(5) $$HD_{95}=\max\left(\underset{p\in P}{\max}\;\underset{g\in G}{\min}\;d\left(p,g\right),\underset{g\in G}{\max}\;\underset{p\in P}{\min}\;d\left(p,g\right)\right)$$

- ASSD: Measures the average distance from the surface of the automated segmentation to the surface of the manual annotation, and vice versa. A lower ASSD demonstrates that the predicted contour closely adheres to the original label.

(6) $$ASSD=\frac{\sum_{p\in P}d\left(p,G\right)+\sum_{g\in G}d\left(g,P\right)}{\left|P\right|+\left|G\right|}$$

Statistical analysis was performed at the case level, utilizing the aggregated predictions from the 5-fold cross-validation. Continuous performance metrics (DSC, HD95, ASSD) are expressed as mean ± standard deviation across the individual patient cases. To evaluate the statistical significance of performance differences between the baseline architectures and the fine-tuned nnU-Net, paired case-level comparisons were conducted using the non-parametric Wilcoxon signed-rank test. To control the family-wise error rate arising from multiple comparisons (three baseline models versus the fine-tuned model), the Holm–Bonferroni correction was applied to all reported *p*-values.

Because distance metrics are undefined for empty sets, the spatial evaluations (DSC, HD95, ASSD) were exclusively calculated on the cohort of 284 histologically confirmed lesion-positive NSCLC cases. To evaluate the model’s pathological detection capabilities and its performance in regions of physiological FDG uptake, voxel-level Sensitivity (Recall) and Precision (Positive Predictive Value) were defined as follows:

(7) $$Sensitivity=\frac{TP}{TP+FN}$$

(8) $$Precision=\frac{TP}{TP+FP}$$

where *TP* (True Positive) represents correctly identified tumor voxels, *FN* (False Negative) indicates missed tumor voxels, and *FP* (False Positive) signifies healthy tissue incorrectly classified as tumor.

Furthermore, the 116 negative-control scans (empty-mask cases) were analyzed independently to determine the physiological False-Positive Rate (*FPR_case_*) at the case level. A negative control scan was classified as a false positive if the automated framework predicted a lesion volume strictly greater than zero after post-processing. The formulation is defined as:

(9) $${FPR}_{case}=\frac{N_{FP\text{\_}scan}}{N_{Negative\text{\_}controls}}$$

where *N_FP_scan_* is the number of negative-control scans containing at least one false-positive prediction, and *N_Negative_controls_* is the total number of healthy/benign cases (*n* = 116).

To assess the absolute clinical agreement in MTV quantification between the customized pipeline and the manual STAPLE consensus, the intraclass correlation coefficient (ICC) was computed. Specifically, a two-way random-effects model for absolute agreement of a single rater, denoted as ICC(2,1), was utilized to account for systematic differences, alongside the 95% confidence intervals (95% CI). In this analysis, an ICC value ≥ 0.90 was predefined as indicating excellent absolute agreement for clinical applicability.

## 3. Results

### 3.1. Performance on Internal Dataset

Table 2 summarizes the hardware environment, optimization schedules, convergence criteria, and training efficiency across all evaluated architectures. The baseline nnU-Net v2 and fine-tuned nnU-Net architectures were trained for 1000 and 500 epochs, requiring approximately 16.0 and 10.0 h per fold, respectively. The MONAI-based ResNet-backbone 3D U-Net and Swin UNETR models were both trained for 300 epochs, with a local training time per fold of approximately 12.5 and 24.0 h, respectively.

The results of the 5-fold cross-validation on the Vietnamese patient PET/CT dataset are reported in detail to identify the optimal model architecture. Table 3 presents the quantitative segmentation performance of three deep learning models (trained from scratch), compared with the model employing a transfer learning strategy (fine-tuned from pretrained weights).

As shown in Table 3, the nnU-Net model (pretrained + fine-tuned) achieved a DSC of 83.4 ± 6.5%. Additionally, this model recorded an HD95 of 5.1 ± 3.6 mm and an ASSD of 2.0 ± 2.2 mm. The precision of the fine-tuned architecture was 89.6 ± 8.2%, indicating control over false positives in regions of physiological FDG uptake.

Among the architectures trained from scratch, the baseline nnU-Net v2 yielded a DSC of 76.6 ± 7.5% and HD95 of 7.3 ± 8.6 mm, followed by Swin UNETR with a DSC of 71.2 ± 8.6%. Although its DSC was lower than that of nnU-Net, Swin UNETR recorded the highest sensitivity (80.4 ± 8.1%). This capability to minimize false negatives can be attributed to the self-attention mechanism inherent to Transformer networks, which facilitates global context modeling. Conversely, the ResNet-backbone 3D U-Net achieved a DSC of 65.0 ± 10.2% and an HD95 of 15.8 ± 8.5 mm.

**Figure 3:**
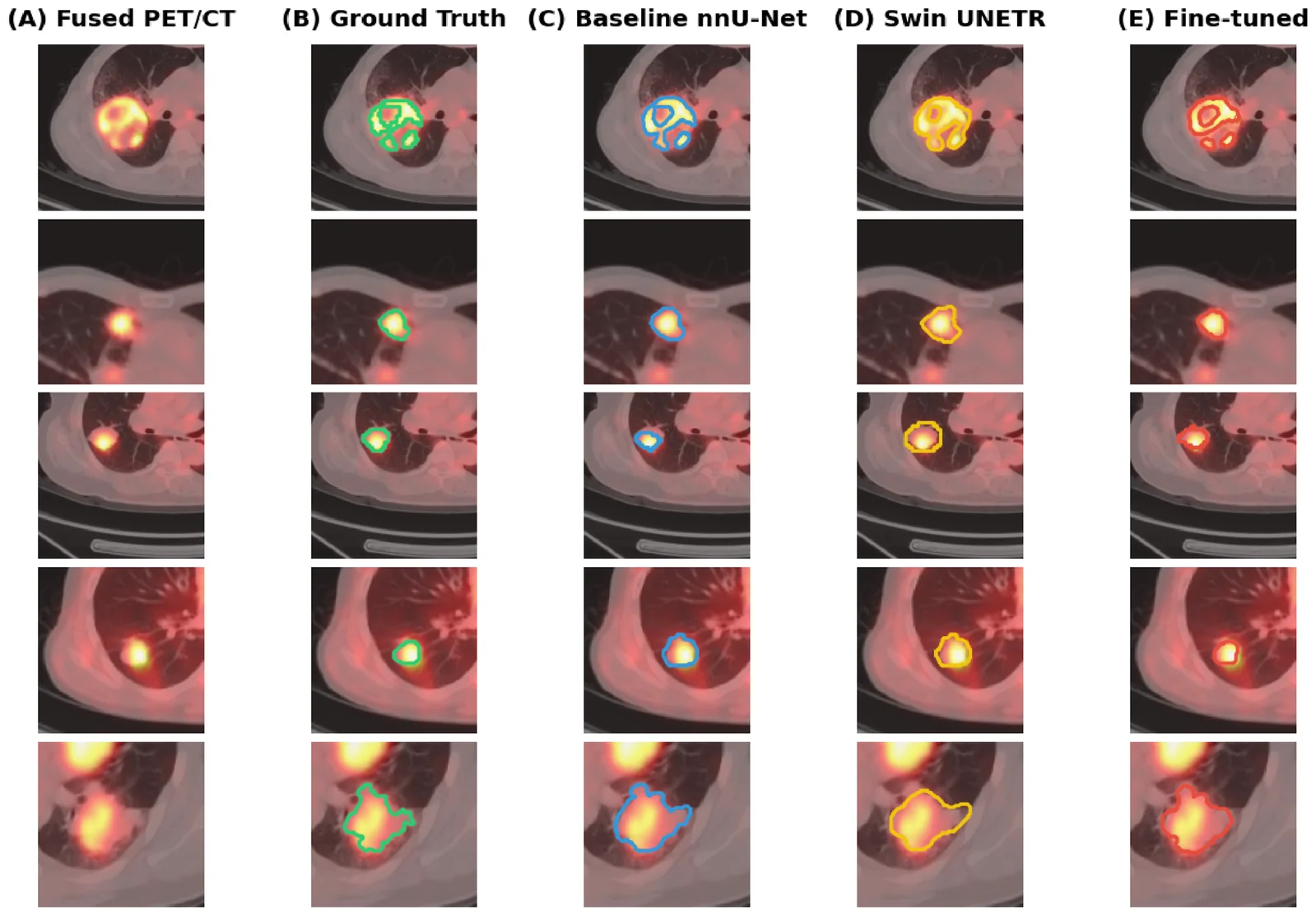
Representative qualitative results with different training architectures. (**A**) Fused PET/CT; (**B**) Ground Truth (STAPLE); (**C**) nnU-Net (Baseline, blue color); (**D**) Swin UNETR (Yellow color); and (**E**) Fine-tuned nnU-Net (Red Color).

Figure 3 presents qualitative results from five representative patient cases, comparing the segmentation outputs of the evaluated architectures. Visual assessment indicates that the baseline nnU-Net and Swin UNETR models exhibit deviations when delineating complex tumor boundaries. In contrast, the fine-tuned nnU-Net demonstrates the closest overlap with the STAPLE consensus, particularly in regions of heterogeneous FDG uptake. Given its higher spatial accuracy and precision, the fine-tuned nnU-Net was utilized for all downstream analyses in this study.

### 3.2. Inter-Observer Agreement

Evaluating a deep learning model against human inter-observer variability is a prerequisite for determining its feasibility for clinical application. In this study, the human baseline, measured as the average pairwise DSC among the three independent nuclear medicine experts, was 80.2 ± 6.6%.

The fine-tuned nnU-Net achieved its highest spatial agreement (DSC = 83.4%) when evaluated against the STAPLE consensus label, as this probabilistic map served as its training target. To evaluate the model’s performance against individual human variability, it was compared with each rater independently. As detailed in Table 4, when evaluated against Rater 1, Rater 2, and Rater 3, the fine-tuned model attained DSC values of 81.5%, 82.2%, and 81.8%, respectively, resulting in an average DSC of 81.8%.

The automated model demonstrated higher spatial agreement with individual physicians than the agreement observed among the physicians themselves. Furthermore, the segmentation performance of the model exceeded the human inter-observer baseline in 68% of the evaluated cases. These findings indicate that the fine-tuned framework captures consensus characteristics and maintains segmentation consistency, mitigating the variability associated with manual delineation. This performance supports its potential application for automated MTV quantification in clinical workflows.

### 3.3. Performance on International Dataset

To evaluate domain generalization and sensitivity to domain shift, the segmentation architectures were applied directly (zero-shot, without retraining) to an independent external dataset: the LUNG-PET-CT-Dx (TCIA) [14]. A sub-cohort of 50 patient cases was selected based on the availability of paired whole-body 18F-FDG PET and unenhanced CT scans, along with primary lung cancer diagnoses (predominantly non-small cell lung cancer). The reference masks for this external cohort were derived from the original dataset annotations provided by thoracic radiologists. To ensure methodological consistency and prevent data leakage, the preprocessing pipeline for the external dataset replicated the procedure applied to the internal validation set.

As presented in Table 5, the models trained from scratch on the internal dataset exhibited performance degradation when evaluated on external data. The baseline nnU-Net v2 and Swin UNETR models recorded average DSC decreases of 22.9% and 21.3%, respectively. Furthermore, these baseline architectures demonstrated substantial spatial boundary errors, with HD95 values reaching 19.9 mm and 21.7 mm, and ASSD values of 7.0 mm and 8.4 mm. Their precision metrics dropped to 65.1% and 69.5%, indicating an increase in false-positive predictions on the external domain. This performance decline originates from systematic differences in scanner protocols, SUV normalization techniques, and demographic variations between Asian and Western patient populations.

Conversely, the fine-tuned nnU-Net model maintained a DSC of 79.8% on the LUNG-PET-CT-Dx dataset, limiting the average drop to 4.5%. The customized pipeline also preserved spatial boundary accuracy, recording an HD95 of 6.9 mm and an ASSD of 2.2 mm. With a precision of 83.7%, the fine-tuning strategy demonstrated a consistent capability to control false positives and adapt to domain shifts compared to the architectures trained from scratch.

**Figure 4:**
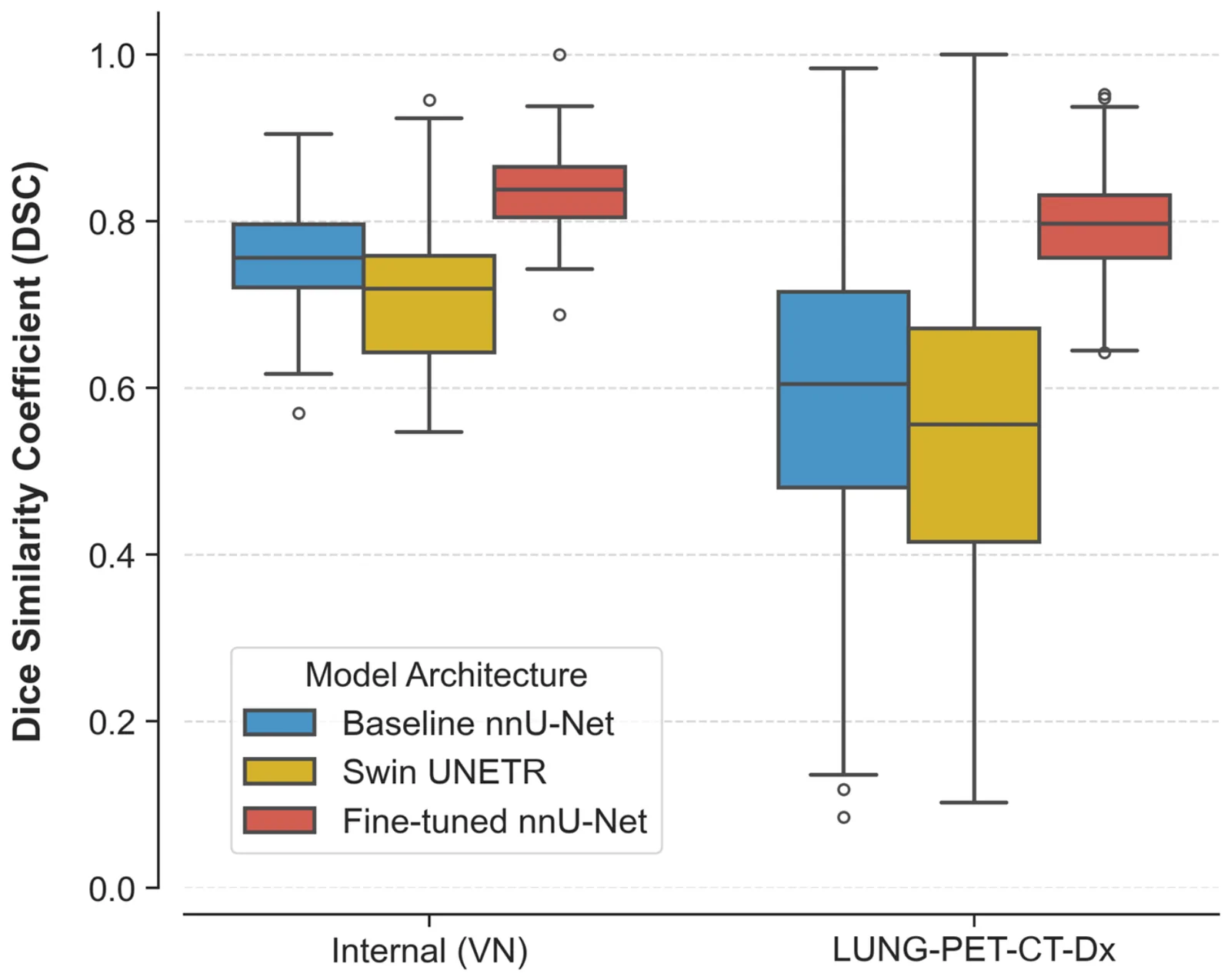
Distribution of DSC across internal and external datasets. The boxplots illustrate the median, interquartile range (IQR), and outlier circles for the benchmarked models, highlighting the superior stability of the fine-tuned framework under domain shift.

The generalization capability of the fine-tuned model is illustrated through the statistical distribution in Figure 4. The chart indicates that when evaluated on external datasets, the baseline architectures (nnU-Net v2 and Swin UNETR) exhibited decreases in median DSC values and increased data dispersion. Specifically, the interquartile ranges (IQRs) for these models widened, accompanied by multiple outliers with performance dropping below a DSC of 0.4. In contrast, the fine-tuned nnU-Net model maintained consistent performance. The IQR of the fine-tuned model on LUNG-PET-CT-Dx datasets remained narrow and comparable to its distribution on the internal dataset. This suggests that the fine-tuned framework preserves segmentation accuracy and mitigates errors associated with variations in imaging protocols and scanner equipment.

### 3.4. Ablation Study: Effect of Pretrained Fine-Tuning

To systematically isolate the empirical benefits of domain-specific fine-tuning from the inherent capabilities of the pretrained model, an ablation study was conducted. We compared three distinct configurations: a baseline model trained from scratch with random initialization, a zero-shot pretrained model (initialized with AutoPET II weights but without any local retraining), and the fine-tuned model in Table 6.

As presented in Table 6, the zero-shot pretrained model yielded a DSC of 76.7%, which is comparable to the baseline model trained entirely from scratch (76.6%). However, a discrepancy was observed in the spatial boundary error. The zero-shot pretrained model exhibited an HD95 of 198.1 mm, compared to 7.30 mm for the baseline model. This spatial error indicates the presence of a domain shift between the training and target datasets. When directly applying the AutoPET II pretrained weights to the local dataset, the model produced distant false-positive predictions (likely in regions of physiological FDG uptake such as the brain, myocardium, or urinary tract). This is driven by systematic differences in scanner protocols, SUV normalization techniques, and the specific focus on NSCLC versus a multi-cancer cohort.

The fine-tuned nnU-Net increased the spatial overlap to a DSC of 83.4% and reduced the HD95 to 5.10 mm. These results reveal that deploying global pretrained weights without local adaptation is susceptible to domain shift artifacts. Furthermore, these findings indicate that an appropriate fine-tuning strategy on the target domain enables the model to adapt to institution-specific imaging protocols, data characteristics, and disease-related variations.

### 3.5. Clinical Assessment of Metabolic Tumor Volume

Beyond spatial overlap metrics (such as DSC and HD95), the clinical utility of a deep learning model in oncology depends on its accuracy in extracting quantitative parameters, particularly the MTV. To assess this capability, the tumor volumes predicted by the automated model were compared with the volumes derived from the STAPLE consensus labels.

**Figure 5:**
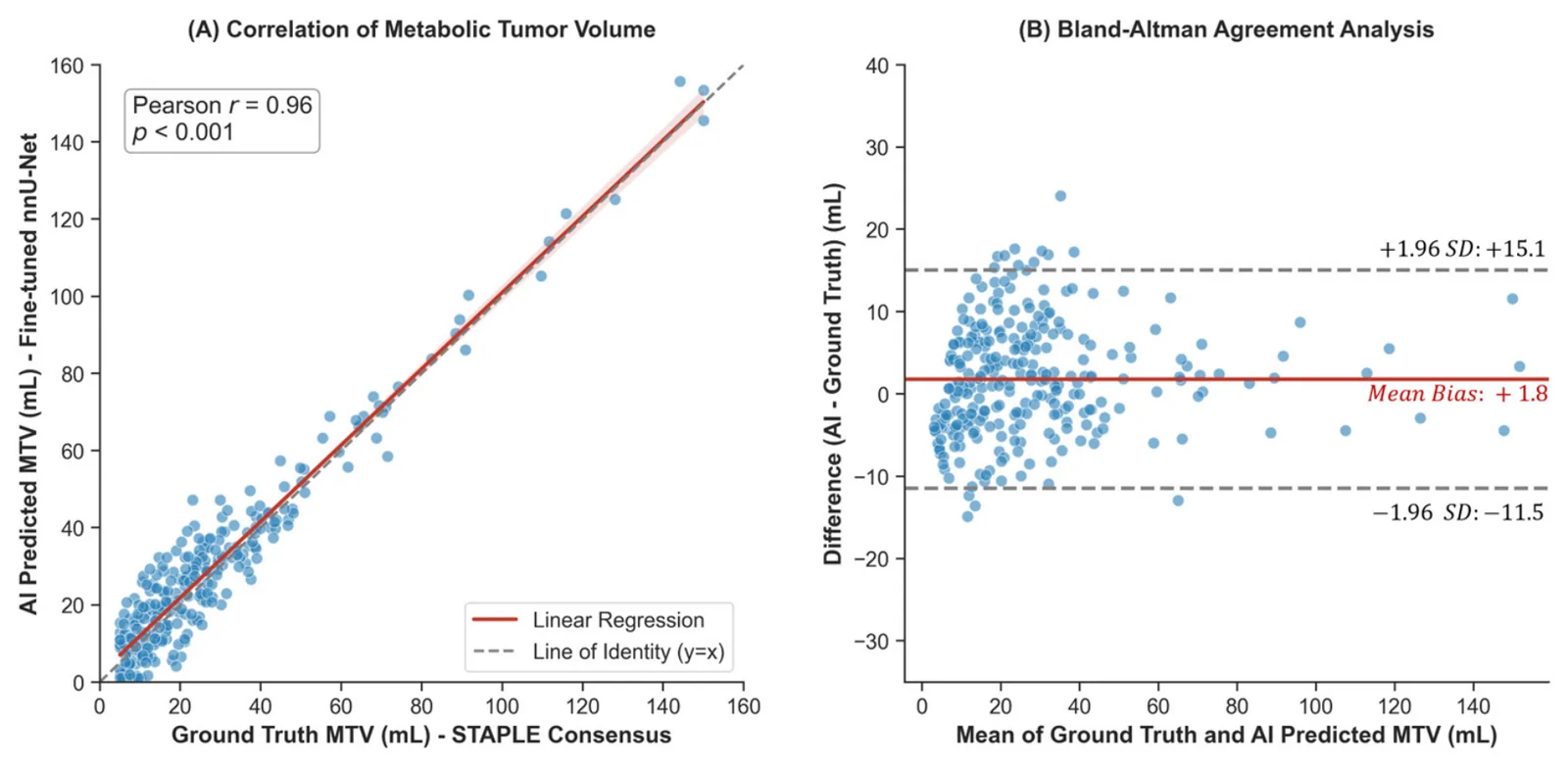
Clinical agreement analysis of MTV extracted by the fine-tuned nnU-Net model versus the expert STAPLE consensus. (**A**) Correlation of metabolic tumor volume and (**B**) Bland–Altman agreement analysis.

Figure 5A presents the statistical analysis evaluating MTV on a per-patient basis (n = 284). The automated predictions demonstrated absolute agreement with the STAPLE consensus, yielding an ICC of 0.94 (95% CI: 0.91–0.96, *p* < 0.001), and a Pearson correlation of *r* = 0.96.

Furthermore, the Bland–Altman analysis [21] (Figure 5B) illustrates the volumetric consistency of the evaluated framework, recording a mean bias of +1.8 mL. The 95% limits of agreement ranged from −11.5 mL to +15.0 mL. Previous multi-center studies evaluating inter-observer variability in NSCLC PET/CT segmentation [22,23] indicate that manual MTV delineations exhibit volume variations of up to 15–20 mL due to the ambiguous boundaries of heterogeneous tumors. Consequently, the volume differences produced by the fine-tuned pipeline fall within the established range of human inter-observer variability, supporting its application for quantitative tumor staging.

The fine-tuning process on the internal data mitigated false-positive phenomena in regions of physiological FDG uptake. Compared to the baseline nnU-Net model, the misidentification rate in areas such as brown fat and the myocardium decreased from 14% to 6%. This reduction indicates that the model differentiated pathological signals from the patient-specific physiological metabolic activities.

**Figure 6:**
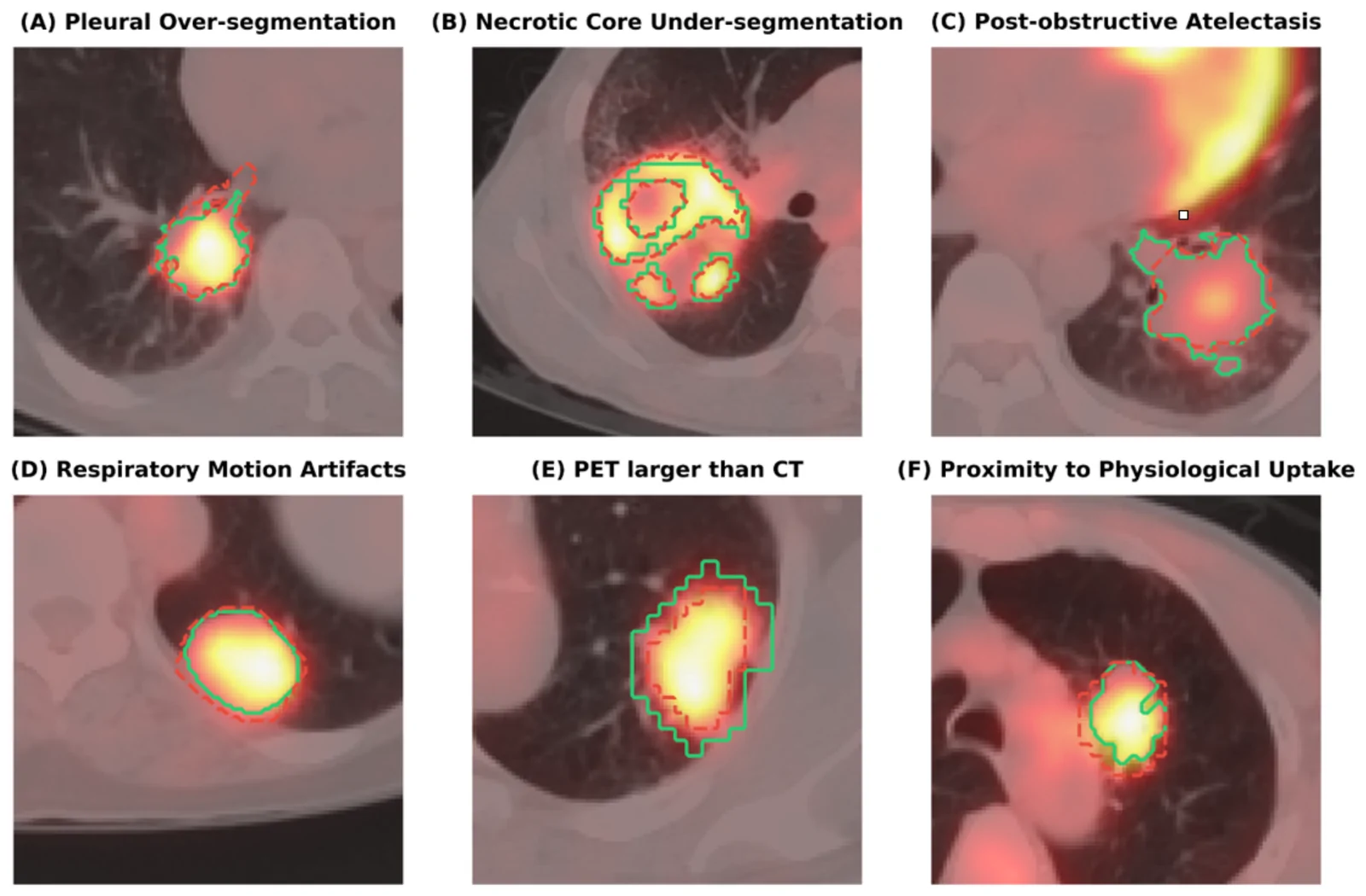
Qualitative analysis of segmentation discordance in challenging anatomical boundary regions: (**A**) pleural over-segmentation; (**B**) under-segmentation of a non-FDG-avid necrotic tumor core; (**C**) Over-segmentation into adjacent post-obstructive atelectasis; (**D**) Boundary deviation affected by spatial misalignment from respiratory motion artifacts; (**E**) Under-segmentation resulting from spatial mismatch; (**F**) Over-segmentation due to the tumor’s close proximity to physiological uptake.

Figure 6 illustrates the visual discrepancies between the STAPLE consensus label (solid green line) and the prediction from the evaluated framework (dashed red line). The model exhibited segmentation differences in complex anatomical and physical scenarios. In instances of close proximity, such as tumor–pleural interfaces, the model produced over-segmentation due to partial volume effects blurring the boundaries (Figure 6A). Figure 6B presents a case of under-segmentation in a tumor containing a non-FDG-avid necrotic core, where the prediction was confined to the hypermetabolic rim and omitted the internal necrotic volume delineated by the expert consensus. Furthermore, the model demonstrated over-segmentation by including adjacent post-obstructive atelectasis, as it struggled to differentiate between the malignant lesion and inflamed tissue on the multimodal images (Figure 6C). Figure 6D shows boundary deviation caused by spatial misalignment from patient respiratory motion artifacts. Finally, Figure 6E highlights under-segmentation resulting from the spatial mismatch where the metabolic PET signal spills over the CT anatomical boundary. These discordant cases illustrate the ongoing challenges in segmenting heterogeneous tumors and handling multimodal imaging artifacts. Finally, Figure 6F demonstrates boundary deviation when the tumor is located in close proximity to regions of high physiological 18F-FDG uptake, which complicates the delineation of the true metabolic tumor volume.

## 4. Discussion

This study implemented and validated an automated NSCLC tumor segmentation framework on multimodal ^18^F-FDG PET/CT images based on nnU-Net v2. The pipeline was optimized for a Vietnamese patient population using a dataset of 400 cases featuring histologically confirmed lesions and STAPLE consensus labels from three nuclear medicine experts. The quantitative results provide evidence of the fine-tuned nnU-Net architecture’s improved performance over architectures trained from scratch, as well as its generalization capability on external data.

In the internal 5-fold cross-validation, the fine-tuned nnU-Net model achieved a DSC of 83.4 ± 6.5%, an HD95 of 5.1 ± 3.6 mm, and a precision of 89.6 ± 8.2%. Compared to the baseline nnU-Net v2 (DSC 76.6 ± 7.5%; HD95 7.3 ± 8.6 mm), the fine-tuned architecture improved spatial accuracy and precision while reducing surface error (ASSD decreased from 2.5 ± 2.3 mm to 2.0 ± 2.2 mm). This aligns with the recommendations of Isensee et al. [16] regarding the benefits of fine-tuning nnU-Net on target data and is consistent with recent studies on domain adaptation in PET/CT segmentation [17,24].

The cross-domain generalization potential was evaluated directly on external datasets. While the pairwise DSC among the three physicians was 80.2%, the fine-tuned model achieved an average DSC of 81.8% when evaluated independently against each rater, peaking at 83.4% against the STAPLE consensus label. The automated performance exceeded the inter-observer baseline in 68% of the cases, supporting the clinical feasibility of the framework. Furthermore, models trained from scratch (baseline nnU-Net v2 and Swin UNETR) exhibited average DSC decreases of 22.9% and 21.3% on the external data, respectively. In contrast, the fine-tuned nnU-Net model recorded a performance decrease of only 4.5% (maintaining a DSC of 79.8% on LUNG-PET-CT-Dx). The statistical distribution illustrates that the fine-tuned model possesses a narrow dispersion range and fewer outliers, mitigating domain shift caused by variations in demographics, SUV distributions, and scanner protocols.

The ablation analysis indicates the value of transfer learning. The zero-shot model yielded a high spatial error (HD95 of 198.1 mm) because the AutoPET II pretrained weights were derived from a multi-cancer cohort. Consequently, the model lacked specificity for lung-confined lesions and was sensitive to variations in local SUV normalization parameters. Transfer learning through local fine-tuning resolved this issue by adapting the network weights to the lung-specific evaluation context. This adaptation successfully reduced the HD95 to 5.1 mm, increased the DSC from 76.6% to 83.4%, and reduced local training time by 37.5%. These findings confirm that deploying globally pretrained weights without local adaptation is susceptible to domain shift artifacts, and that target-domain fine-tuning is necessary to ensure reliable automated segmentation.

Regarding clinical utility, the fine-tuned model demonstrated a linear correlation with the MTV manually measured by experts (Pearson r = 0.96, *p* < 0.001), with a Bland–Altman mean bias of +1.8 mL (95% LoA: −11.5 to +15.0 mL). Concurrently, the false-positive rate in regions of physiological FDG uptake (such as brown fat, myocardium, and brain) decreased from 14% to 6%, indicating that the model differentiated pathological signals from physiological metabolic activities.

Despite the overall segmentation accuracy, qualitative analysis (Figure 6) revealed limitations in complex anatomical boundaries. As observed in Figure 6A, the model occasionally exhibited deviations in delineating the boundary between the tumor and adjacent structures such as the pleura, leading to over-segmentation. This is likely exacerbated by the partial volume effect inherent to PET imaging, which blurs signals at the edges. Conversely, for large tumors with central necrosis (Figure 6B), the network tended toward under-segmentation. Because the necrotic region lacks FDG avidity, the model primarily relied on the hypermetabolic PET signal, resulting in the omission of the tumor core despite the CT channel providing tissue density information. These localized boundary discordances explain the residual error in the DSC (~16.5%) and HD95 (5.1 mm) metrics. Future algorithmic refinements could focus on integrating boundary-aware loss functions or enhancing attention mechanisms for the CT feature stream, compelling the model to recognize morphology comprehensively rather than relying predominantly on FDG metabolism.

To systematically quantify the limitations observed in Figure 6, an error analysis was conducted on the discordant cases (defined as predictions yielding a DSC < 0.70). Among these suboptimal segmentations, pleural over-segmentation (Figure 6A) was the most frequent error, accounting for 45% of the failed cases. This error was predominantly associated with lesions located directly adjacent to the chest wall or mediastinum, where partial volume effects blur the anatomical borders. Under-segmentation of necrotic cores (Figure 6B) constituted 23% of the errors, primarily observed in large-volume tumors (volume > 43 mL) exhibiting central necrosis and non-avid ^18^F-FDG centers. Furthermore, the remaining discordant cases were primarily distributed among three specific challenging scenarios. Over-segmentation into adjacent post-obstructive atelectasis (Figure 6C) accounted for 12% of the errors, as the model struggled to differentiate malignant tissue from secondary lung inflammation. Boundary deviations caused by spatial misalignment from respiratory motion artifacts (Figure 6D) represented 12%. About 8% of the errors were linked to under-segmentation resulting from the blooming effect, where the metabolic PET signal spills over the CT anatomical boundary (Figure 6E). These numerical findings indicate that the evaluated framework faces specific challenges when processing tumors with extreme morphological heterogeneity, adjacent inflammatory processes, or multimodal spatial mismatches.

The evaluation of external datasets was conducted in silico, and the direct impact on patient prognosis was not measured. Future prospective, multi-center trials are required to establish the clinical feasibility and generalization capacity of this automated framework in real-world nuclear medicine and clinical oncology workflows.

Additionally, while the local fine-tuning phase required 10.0 h per fold, this efficiency metric inherently relies on the substantial computational cost previously amortized during the generation of the public AutoPET II weights. A potential limitation of this comparative analysis is the uneven training budget. Specifically, the MONAI-based models (ResNet-backbone 3D U-Net and Swin UNETR) were trained for 300 epochs until early-stopping convergence, whereas the nnU-Net architecture ran for a default budget of 1000 epochs. Although this discrepancy reflects the utilization of native optimal configurations for each framework, it results in unequal computational budgets. Future benchmarking studies should consider standardizing these computational parameters to ensure a strictly equitable comparison baseline.

## 5. Conclusions

This study investigated an automated NSCLC tumor segmentation framework for ^18^F-FDG PET/CT based on a fine-tuned nnU-Net v2 architecture using a histologically confirmed dataset of 400 Vietnamese patients. Compared with models trained from scratch, including the baseline nnU-Net and Swin UNETR, the fine-tuned model achieved improved segmentation performance, demonstrated strong agreement with expert-derived MTV measurements (r = 0.96), and reduced physiological false-positive detections from 14% to 6%.

The evaluated framework also showed competitive performance on an external dataset, achieving a DSC of 79.8% on LUNG-PET-CT-Dx. Compared with the baseline model, the fine-tuned approach exhibited substantially lower performance degradation under domain shift conditions, with an average DSC decrease of 4.5% versus 22.9% for the baseline model. In addition, the fine-tuning strategy reduced local training time by 37.5%, improving computational efficiency.

These findings suggest that domain shift may present a bidirectional challenge for PET/CT tumor segmentation, affecting both locally trained models applied to external datasets and globally pretrained models applied to local datasets. The results further suggest that target-domain fine-tuning can improve model adaptation to differences in imaging protocols, scanner characteristics, and disease distributions, which may facilitate automated MTV quantification. Nevertheless, further independent multi-center and prospective validation will be necessary to confirm the generalizability and broader clinical applicability of the proposed framework.

## Figures and Tables

**Table 1:** Clinicopathological characteristics of the patient cohort and PET/CT dataset.

Parameter	Values
Total number of cases analyzed	400 cases
+NSCLC cases (histologically confirmed)	284 cases
+Negative controls	116 cases
Dice score (DSC) of 3 Raters	80.2 ± 6.6%
Mean age (years)	61.2 ± 9.9
Gender (Male/Female)	228/172
Lesion Characteristics	
+Total NSCLC lesions	342
+Mean tumor size (cm)	2.8 ± 1.0
Histological Subtype	
+Adenocarcinoma	198 cases (69.7%)
+Squamous cell carcinoma	86 cases (30.3%)
Mean SUVmax by Stage	
+Stage I–II	4.2 ± 1.8
+Stage III–IV	7.4 ± 3.1

**Table 2:** Hardware, optimization schedules, and computational cost across benchmarked architectures.

Model	Hardware	Epoch Budget	Optimizer & Schedule	Convergence Criteria	Local Training Time (per Fold)
nnU-Net v2 (baseline)	1× RTX Pro 6000	1000	SGD, polyLR decay	Exhaustion of learning rate schedule	~16.0 h
ResNet-backbone 3D U-Net (MONAI)	1× RTX Pro 6000	300	AdamW, Cosine Annealing	Validation pseudo-Dice early stopping	~12.5 h
Swin UNETR	1× RTX Pro 6000	300	AdamW, Cosine Annealing	Validation pseudo-Dice early stopping	~24.0 h
Fine-tuned nnU-Net	1× RTX Pro 6000	500	SGD, polyLR decay	Exhaustion of learning rate schedule	~10.0 h

**Table 3:** Quantitative comparison of segmentation performance across different deep learning architectures on the internal Vietnamese cohort.

Model	DSC (%) ↑	HD95 (mm) ↓	ASSD (mm) ↓	Sensitivity (%) ↑	Precision (%) ↑	*p*-Value (vs. Fine-Tuned)
ResNet-backbone 3D U-Net	65.0 ± 10.2	15.8 ± 8.5	6.2 ± 2.1	66.2 ± 10.5	68.1 ± 12.0	<0.001
Swin UNETR	71.2 ± 8.6	9.5 ± 6.2	3.1 ± 1.5	80.4 ± 8.1	77.5 ± 10.2	<0.001
nnU-Net v2	76.6 ± 7.5	7.3 ± 8.6	2.5 ± 2.3	77.2 ± 7.4	81.3 ± 8.5	<0.01
nnU-NET (pretrained + fine-tuned)	83.4 ± 6.5	5.1 ± 3.6	2.0 ± 2.2	79.2 ± 8.2	89.6 ± 8.2	-

Note: The symbol ↑ indicates higher is better; ↓ indicates lower is better.

**Table 4:** Agreement DSC analysis between the fine-tuned model and individual expert radiologists.

Model	Rater 1	Rater 2	Rater 3	STAPLE Consensus
nnU-Net (pretrained + fine-tuned)	81.5%	82.2%	81.8%	83.4%

**Table 5:** Zero-shot generalization performance of the segmentation models on the LUNG-PET-CT-Dx dataset.

Model	DSC (%)	HD95 (mm)	ASSD (mm)	Precision (%)	Average Drop (DSC)
nnU-Net v2 (baseline)	59.8	19.9	7.0	65.1	−22.9%
Swin UNETR	56.0	21.7	8.4	69.5	−21.3%
Fine-tuned nnU-Net	79.8	6.9	2.2	83.7	−4.5%

**Table 6:** Ablation study results evaluating the impact of transfer learning on segmentation accuracy and training efficiency.

Configuration	Initialization	DSC	HD95 (mm)
nnU-Net v2 (baseline)	Random Weights	76.6%	7.30
Zero-shot Pretrained	AutoPET II Weights	76.7%	198.1
Fine-tuned nnU-Net	AutoPET II Weights	83.4%	5.10

## Data Availability

The raw whole-body 3D PET/CT imaging datasets analyzed in this study are not publicly available due to institutional regulations and ethical restrictions related to patient privacy. De-identified datasets may be available from the corresponding author upon reasonable request and subject to approval by the Institutional Review Board of Ho Chi Minh Oncology Hospital, Vietnam.
